# RETRACTED ARTICLE: Causes of death in patients with a history of severe anorexia nervosa

**DOI:** 10.1186/s40337-022-00716-5

**Published:** 2022-12-24

**Authors:** Patricia Westmoreland, Alan Duffy, Renee Rienecke, Daniel Le Grange, Thomas Joiner, Jamie Manwaring, Ashlie Watters, Philip Mehler

**Affiliations:** 1 Eating Recovery Center, Denver, CO USA; 2 ACUTE Center of Eating Disorders, Denver, CO USA; 3 University of Colorado School of Medicine, Denver, CO USA; 4 Department of Psychiatry and Behavioral Sciences, University of California, San Francisco, CA USA; 5 Department of Psychology, Florida State University, Tallahassee, FL USA; 6 Department of Psychiatry and Neuroscience, The University of Chicago, Chicago, IL USA; 7 Department of Psychiatry and Behavioral Sciences, Northwestern University, Chicago, IL USA

The authors have retracted this article because it contains identifying information about individual patients included in their analysis. The content of this article is no longer available online in order to protect patient confidentiality. The authors have been offered the opportunity to submit an appropriately amended version of their manuscript to the journal.

Alan Duffy has stated on behalf of all authors that they agree with this retraction.

